# Hydrophilicity as Compensation of Hydrogen-Bond Defects at Graphene Oxide Interfaces

**DOI:** 10.1021/acsnanoscienceau.6c00063

**Published:** 2026-07-20

**Authors:** Patrick R. B. Côrtes, Nicolás A. Loubet, Mateus H. Köhler, José Rafael Bordin, Gustavo A. Appignanesi

**Affiliations:** † Departamento de Física, Instituto de Física e Matemática, 37902 Universidade Federal de Pelotas, 96010-610 Pelotas, Brazil; ‡ INQUISUR, Departamento de Química, 28249 Universidad Nacional Del Sur (UNS)-CONICET, Bahía Blanca B8000, Argentina; ¶ Departamento de Física, 28118 Universidade Federal de Santa Maria, 97105-900 Santa Maria, Brazil; § Fachbereich Physik, Universität Konstanz, 78464 Konstanz, Germany

**Keywords:** Water, Graphene Oxide, Solid−liquid interface, Molecular Dynamics, Hydrogen bond

## Abstract

Hydrophilicity is well-defined macroscopically, yet its molecular origin remains elusive. Here, molecular dynamics simulations combined with the V_4S_ structural index are used to resolve hydrophilicity at the molecular-site level and quantify the spatial extent of hydration perturbations induced by oxygen functional groups at graphene oxide interfaces. We show that epoxy and hydroxyl groups induce distinct hydration regimes: hydroxyl groups form strong hydrogen bonds and stabilize the interfacial network over larger spatial ranges, whereas epoxy groups promote weaker, distorted interactions that disrupt local connectivity. This contrast demonstrates that chemically similar functionalities can generate qualitatively different interfacial responses, highlighting the central role of nanoscale heterogeneity. Within this framework, hydrophobicity emerges as the inability of a surface to compensate for hydrogen-bond defects in its hydration layer, providing a microscopic basis for wettability and guiding the design of carbon-based nanomaterials.

## Graphical abstract

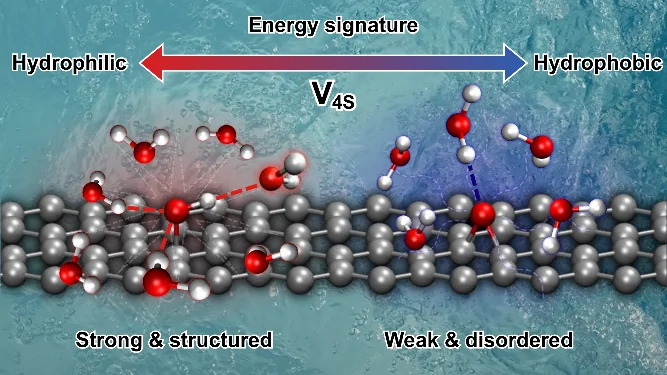

## Introduction

Graphene, a one-atom-thick two-dimensional material with remarkable mechanical, electrical, and thermal properties, has been the subject of extensive research since its experimental isolation.^1,2^ Despite these advantages, its intrinsic hydrophobicity and poor dispersibility in aqueous media limit its applicability in areas where water–surface interactions are central, such as membrane separations, catalysis, and biointerfaces.^3^ In this context, graphene oxide (GO) has emerged as a chemically versatile derivative. The incorporation of oxygen-containing functional groups—primarily hydroxyl (−OH), epoxide (-O−), and carboxyl (−COOH)—confers hydrophilicity and enables stable dispersion in water.^4,5^ These functionalities create chemically heterogeneous surfaces composed of distinct local environments, underpinning the use of GO in applications ranging from water purification and desalination^6^ to sensing and energy storage.^7–9^

The interaction of water with graphene-based materials plays a central role in determining their physicochemical properties and performance. Interfacial hydration influences key features such as interlayer spacing, permeability, adsorption, and surface reactivity.^10–14^ In particular, recent studies have highlighted the importance of nanoscale water structuring in controlling adsorption processes in carbon nanomaterials. For instance, hydration layers formed around carbon nanotubes have been shown to significantly modulate the adsorption of pharmaceutical contaminants by affecting both the accessibility of adsorption sites and the associated energy barriers.^15^ Similarly, in nanoconfined environments, the interplay between oxidation and confinement in graphene-based systems strongly influences the hydrogen-bond (HB) network, water structuring, and transport properties, with direct implications for membrane performance and water treatment technologies.^16^

At the microscopic level, water interactions with GO are inherently site-dependent. Hydroxyl groups can directly participate in hydrogen bonding, whereas epoxide groups interact through a combination of electrostatic and dispersion contributions.^17,18^ In addition, oxygen functionalities tend to form nanoscale clusters, leading to heterogeneous surfaces composed of oxidized domains interspersed with graphitic regions.^19,20^ This chemical patterning has been linked to the coexistence of hydrophilic character and fast water transport in GO-based membranes.^11,21^ However, while these studies establish the importance of chemical heterogeneity, a quantitative, site-resolved description of how different functional groups modulate water structure and energetics remains elusive.

This issue is particularly relevant because interfacial water is not merely a passive probe of surface chemistry. In bulk water, the local structure is governed by a fluctuating hydrogen-bond network in which each molecule tends to adopt a nearly tetrahedral environment. When water approaches a surface, this balance is locally perturbed: one or more hydrogen-bonding directions may become unavailable, and the surface must compensate for the resulting energetic defect through sufficiently favorable water–surface interactions. Therefore, the hydrophilicity of a chemically heterogeneous surface cannot be fully understood solely from the presence of polar groups, but rather from the local balance among hydrogen-bond disruption, water–surface compensation, and the spatial propagation of this perturbation.^22–24^

A central difficulty, therefore, lies in the characterization of hydrophilicity at the molecular scale. Macroscopic descriptors such as the water contact angle provide only averaged information and cannot resolve the spatial variability associated with different functional groups. As a consequence, they do not capture how specific chemical environments locally perturb the hydrogen-bond network of water, nor how these perturbations propagate into the surrounding hydration layer. Consequently, a quantitative description of how specific oxygen functionalities compensate for, or fail to compensate for, local hydrogen-bond defects remains lacking.

Traditional descriptors such as hydrogen-bond counting, tetrahedral order parameters, and radial distribution functions capture local structure but lack a direct energetic interpretation, often classifying weak interactions as hydrogen bonds based solely on geometry.^25,26^ Density-based approaches, while effective in describing hydrophobicity at a collective level, do not resolve the site-specific chemical heterogeneity of functionalized surfaces. Consequently, a unified, spatially resolved framework linking local energetics and hydrogen-bond organization remains underexplored, thereby encouraging the development of new descriptors capable of directly characterizing hydration environments.

In recent work, an energy-based structural indicator of water, denoted V_4S_, has been introduced to characterize local interaction environments in bulk, interfacial, and nanoconfined systems.^27–29^ This index is constructed from the energetics associated with four tetrahedrally arranged interaction sites around a water molecule, enabling a direct assessment of the balance between water–water and water–surface interactions. Within this framework, hydrophobicity can be quantitatively interpreted as the inability of a surface to compensate for the hydrogen-bond defects it induces in its hydration layer. This defines a defect interaction threshold (DIT) of approximately −6 kJ/mol, corresponding to about 30% of a typical HB energy, which coincides with the transition between hydrophilic and hydrophobic regimes (θ ≈ 90°).^29^ By rationalizing the molecular nature of such transition (an issue that remained elusive for a long time), this approach enabled the establishment of absolute values for hydrophobicity. In turn, the modest energetic value highlighted by the DIT leads us to recalibrate usual energetic intuitions since interactions much lower than the energy of a typical hydrogen bond are required in order to ensure (at least partial) hydrophilic behavior. The V_4S_ index provides a parameter-free and spatially resolved description of local hydration environments and has been successfully applied to chemically functionalized surfaces and biomolecular systems.^27–30^ Compared to approaches based on water density fluctuations,^31^ it offers a direct energetic interpretation that can be applied at arbitrary spatial resolution.

In this work, we employ the V_4*S*_ index together with dynamical analyses to investigate how the local hydrophilicity of a graphene sheet is affected by the insertion of epoxy and hydroxyl groups. Using this index, we quantitatively characterize the local energetic impact of the surface on water molecules in the hydration layers, as well as the spatial extent of the effects induced by each oxide group. The paper is organized as follows: in the Methods section, we present the simulation and analysis details; this is followed by the Results and Discussion section, and finally by the Conclusions section, which summarizes the main findings.

## Methods

### Simulation Details

Three systems were generated: pristine graphene sheets, and graphene sheets functionalized with epoxy (Figure 1(a)) and hydroxyl (Figure 1(b)) groups. To improve the statistics of interactions, nine sites of the corresponding oxide group were introduced in each functionalized sheet, sufficiently separated to avoid mutual interactions. The simulation box dimensions are 49.00 Å in the *x* direction, 51.12 Å in *y*, and 50 Å in *z*. The graphene sheet, generated using Graphene-GUI,^32^ is periodic in the *xy* plane and positioned at *z* = 25 Å. The force field parameters, including Coulomb and Lennard-Jones (LJ) interactions, were taken from Hummer et al.^33^ and Stauffer et al.^34^ The systems were solvated using the SPC/E water model,^35^ with 3760 molecules for pristine graphene, 3879 for the epoxy-functionalized system, and 3922 for the hydroxyl-functionalized one.

**Figure 1:**
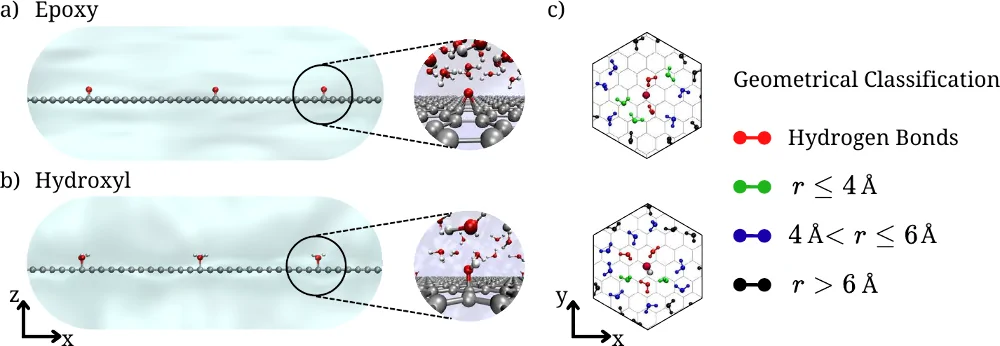
On the left, representations of the simulated systems, showing plates functionalized with epoxy and hydroxyl groups. On the right, an illustration of the geometric classification of water molecules into different analysis groups.

The simulations were performed using the GROMACS molecular dynamics package.^36,37^ The systems were first relaxed through an energy minimization step. To ensure proper density equilibration at 1 bar, we performed alternating equilibration stages: 200 ps in the NVT ensemble, 400 ps in NpT, 200 ps in NVT, and an additional 100 ps in NpT. During the NpT stages, the pressure was coupled to 1 bar, allowing the system to adjust its density to reach a stable state before production. After verifying the convergence of the energy and density, we carried out a 4 ns production run in the NVT ensemble at 300 K. The dynamics were performed using a 1 fs time step. Periodic boundary conditions were applied, and equations of motion were integrated using the Velocity-Verlet algorithm.^38^ The Nosé–Hoover thermostat was employed,^39,40^ Lennard-Jones cross parameters were determined using the Lorentz–Berthelot mixing rules,^41,42^ and long-range interactions were treated using the Particle Mesh Ewald method.^43^

### Analysis

In order to quantify how the introduction of functional groups modifies local hydrophilicity, we employed the V_4S_ index,^27,28^ a recently introduced parameter that has successfully elucidated interfacial interactions between water and other materials.^15,29,30,44^ While fluctuation-based and cavity-formation free-energy descriptors provide a robust thermodynamic framework for hydrophobicity,^45^ they are often limited by their nonlocal nature and reliance on an arbitrarily defined observation volume. In contrast, the V_4S_ index acts as a locally defined, energy-based probe which also has the advantage of providing an absolute scale.^46^

To compute V_4S_, the four interaction sites of a water molecule are first defined as the vertices of a perfect tetrahedron (angle of 109.47°) centered on the oxygen atom and constructed from the hydrogen positions, with each H defining one of the vertices. Next, all heavy atoms within a distance of up to 5 Å from any of these vertices are selected, and each atom is assigned to its nearest vertex. Finally, the interaction energy between the water molecule and each of the resulting groups is calculated and ranked from the lowest (most negative) to the highest value, the latter corresponding to V_4S_. It is important that the V_4S_ calculation is performed using minimized configurations, referred to as inherent dynamics, in order to eliminate fluctuations and thermal noise arising from temperature effects.

To determine how local the effects of the inserted functional group are, at each time step the water molecules were classified into different categories, as shown in Figure 1(c). The classification consists of molecules that form hydrogen bonds (HBs) with the functional group according to a geometric criterion, namely, a maximum O–O distance of 3.5 Å and a maximum H–O–O angle of 30°.^47,48^

The remaining groups consist of molecules belonging to the first hydration layer of the membrane, subdivided as follows: those located at a radial distance from the functional group $\left(r=\sqrt{x^{2}+y^{2}}\right)$ smaller than 4 Å but not forming HBs; those occupying the annular region between 4 Å and 6 Å, and finally those at distances greater than 6 Å, which correspond to a regime analogous to that of pristine graphene.

To better understand how local changes affect the dynamics of water molecules in the vicinity, we determined the hydrogen-bond correlation time τ between water and the functional group. To ensure sufficient temporal resolution for the analysis of hydrogen-bond dynamics, an additional set of simulations was performed, consisting of 100 independent replicas of 50 ps each, with configurations saved every 20 fs. The mean-squared displacement (MSD) was also calculated from this set of simulations. The groups defined in Figure 1(c) were assigned based on the first configuration of each replica. The VMD software^49^ was used for trajectory visualization and image rendering.

## Results and Discussion

In bulk, water molecules are constantly forming and breaking hydrogen bonds. At the molecular scale, water exhibits a mixed behavior, in line with a two-liquid scenario, with molecules forming clusters mediated by these interactions, while others move more freely, diffusing between clusters.^50–53^ A stable and commonly observed configuration is the tetrahedral cluster, in which a central molecule forms hydrogen bonds with four surrounding neighbors. Accordingly, the average number of HBs per molecule is often close to this value.^54^

However, in contact with a surface, many water molecules will inevitably have one of their four interaction sites oriented toward the interface. The hydrophilicity of a material can be associated with its ability to compensate for the hydrogen bonds that water molecules no longer form due to not being in the bulk.^29^ The cutoff value for a hydrogen bond may vary slightly depending on the water model employed, but it is typically close to −12 kJ/mol, already corresponding to a weak/distorted bond. In contrast, a typical well-formed HB has an energy between −20 and −25 kJ/mol.^52,54^

Whether these missing hydrogen bonds can be energetically compensated depends on the interfacial interactions established by the surface. Systems close to the hydrophilic–hydrophobic transition, such as pristine graphene, provide only limited compensation for the missing hydrogen bonds, leading interfacial water to preserve distorted or undercoordinated local arrangements that become directly reflected in the V_4S_ distributions.

The bimodal V_4S_ distribution shown in Figure 2(a) for pristine graphene is consistent with the behavior observed in analogous systems in previous studies.^16,27,28^ The smaller and broader peak, located near the shaded region, corresponds to water–water interactions, whereas the sharper peak, centered around −5 kJ/mol, is associated with water–carbon interactions.

**Figure 2:**
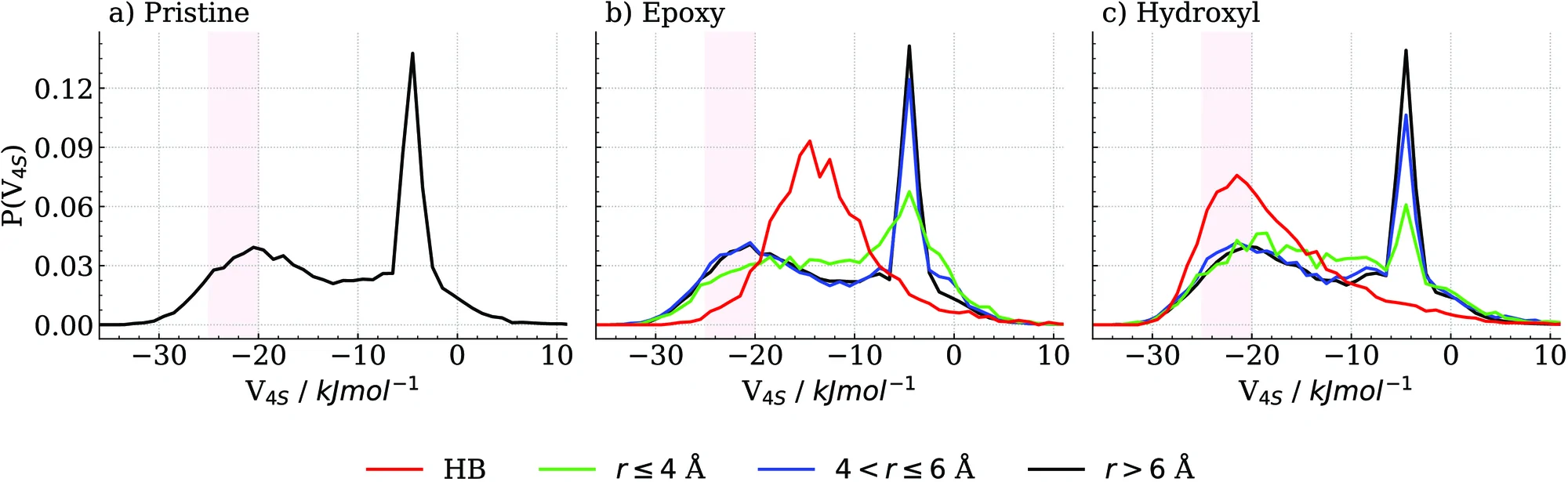
Normalized distribution of V_4S_ values for the three systems studied. From left to right: (a) pristine graphene, (b) epoxy, and (c) hydroxyl. The color code follows that shown in Figure 1. The shaded region indicates values between −20 and −25 kJ/mol, corresponding to energetically well-formed hydrogen bonds.

When analyzing the black curves in the epoxy and hydroxyl panels (Figures 2(b) and 2(c) respectively), which correspond to water molecules at a radial distance greater than 6 Å from the functional group, the behavior, as expected, is similar to that observed for pristine graphene. Moreover, molecules located at distances between 4 and 6 Å, represented by the blue curves, also experience the surface in a manner similar to the pristine case, already indicating that the effects of the functional groups are essentially local.

In contrast, the red group, corresponding to water molecules that form HBs with the functional group, and the green group, consisting of molecules located nearby (*r* < 4 Å) but not forming HBs with the group, exhibit behavior markedly different from that observed for pristine graphene.

Examining the green curve in the epoxy system, one can still observe some similarity to the water–graphene behavior. However, it cannot be described as a bimodal distribution; rather, it displays a single pronounced peak in the hydrophobic region ( >−6 kJ/mol).^29^ In the hydroxyl case, this peak is less pronounced relative to the rest of the distribution. This suggests that hydroxyl groups have a less localized effect, perturbing nearby water molecules that are not engaged in hydrogen bonding. Additional calculations performed with the TIP4*P*/2005 water model and analyses based on both inherent-structure and real-dynamics configurations yielded essentially identical V_4S_ distributions and preserved all qualitative trends discussed herein (see Supporting Information).

The difference in terms of local hydrophilicity becomes evident when analyzing the V_4S_ distributions for molecules forming hydrogen bonds (red curves). Water–epoxy interactions lie in an intermediate energy range, neither as weak as graphene nor as hydrophilic as water–water interactions. This indicates that these are energetically poor HBs, even though they satisfy the geometric criteria. In contrast, the hydroxyl system exhibits a distribution mode coinciding with the energy range of water–water interactions and occupying the shaded region associated with high–quality HBs. Therefore, water–hydroxyl interactions are equivalent to water–water interactions, whereas water–epoxy interactions are significantly weaker.

To further rationalize these observations, it is useful to interpret them within a free-energy framework. From this perspective, interfacial hydration is governed not simply by the strength of water–surface interactions, but by their ability to compensate for hydrogen-bond defects, which defines the local free-energy landscape. When a water molecule approaches the interface, the disruption of hydrogen-bonding directions generates an energetic penalty that must be offset by surface interactions. The V_4S_ index directly quantifies this balance.

Within this framework, the behavior of epoxy groups becomes particularly revealing. Despite introducing polarity at the surface and satisfying geometric hydrogen-bonding criteria, epoxy groups do not provide interactions strong enough to compensate for hydrogen-bond disruption. As a consequence, they induce less hydrophilic local environments, effectively acting as weakly interacting defects in the interfacial network. In contrast, hydroxyl groups effectively compensate for this energetic penalty, stabilizing a locally hydrophilic hydration structure.

This naturally raises the question of the spatial extent of these effects. To address this, we perform a spatially resolved analysis of the V_4S_ index. Figure 3 compares the average V_4S_ values, computed in surface bins for water molecules in the hydration monolayer, for the epoxy and hydroxyl systems. The hydroxyl group induces a much stronger energetic perturbation in the surrounding water structure over a range of approximately 3 Å, whereas the epoxy group produces only minor changes relative to the rest of the monolayer. Together with the distribution of hydrogen positions around the hydroxyl group, this pattern suggests that hydrogen bonds can form in multiple directions at a nearly constant radius.

**Figure 3:**
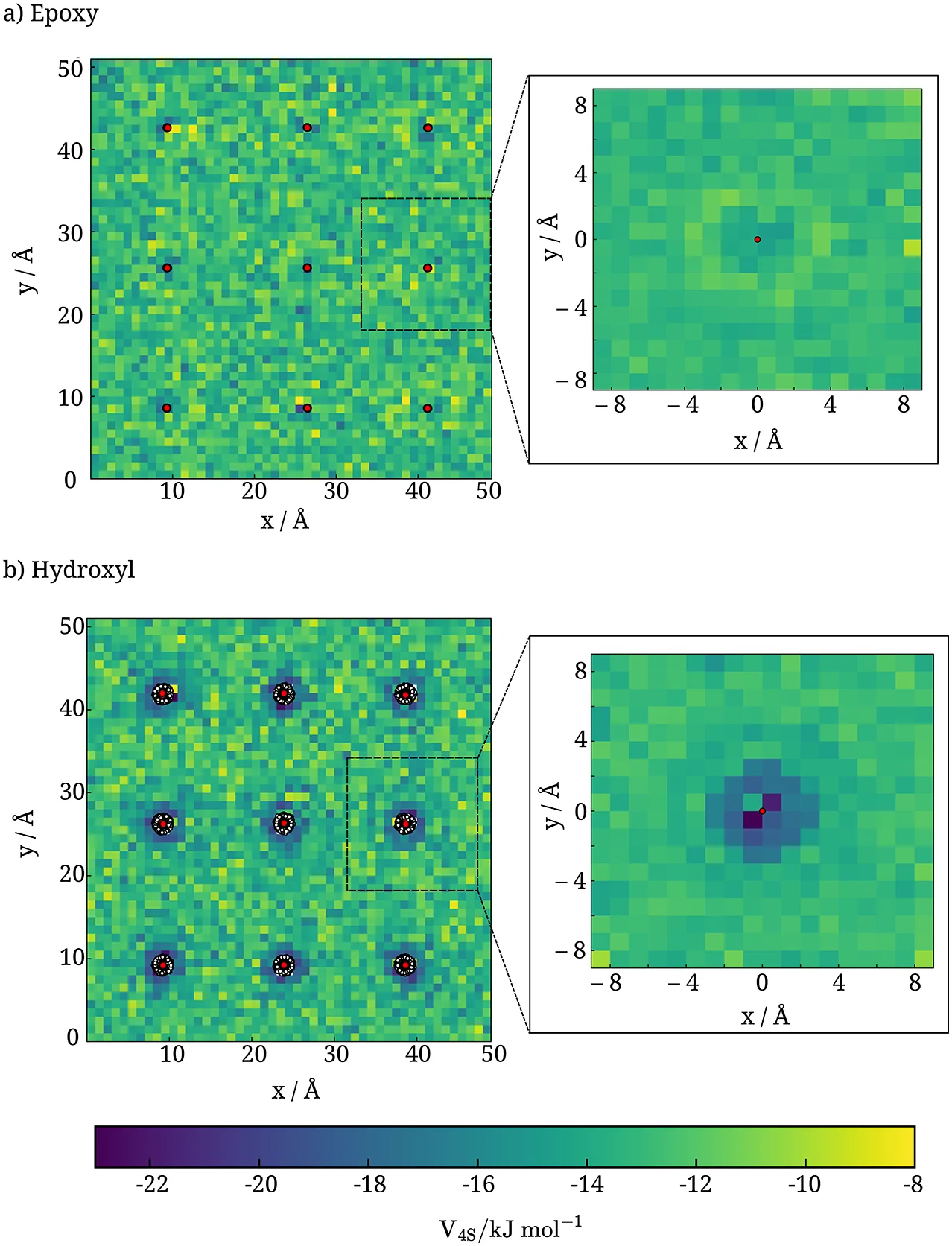
⟨V_4S_⟩ index was computed on a 1 Å × 1 Å grid over the surfaces of the (a) epoxy and (b) hydroxyl systems for the hydration monolayer. The inset shows the nine sites superimposed onto a single position, smoothing local irregularities. Small circles mark the recorded positions of oxygen (red) and hydrogen (white) atoms in the functional groups.

The spatial extent of these perturbations can be interpreted as a characteristic decay length of the interfacial energetic field induced by the functional group. The fact that this length scale remains on the order of a few angstroms indicates that the disruption and compensation of hydrogen bonds are highly localized processes, with limited propagation across the hydration layer.

The effects of hydrogen-bond strength are directly reflected in the dynamics of water molecules surrounding the functional groups. The hydrogen-bond dynamics were characterized through the continuous hydrogen-bond autocorrelation function, f­(t), shown in Figures 4. The resulting autocorrelation functions were fitted using a stretched exponential form,

1 $$f\left(t\right)=\exp\left[-{\left(\frac{t}{\tau }\right)}^{\beta }\right]$$

wher τ is the characteristic relaxation time and β is the stretching exponent.

**Figure 4:**
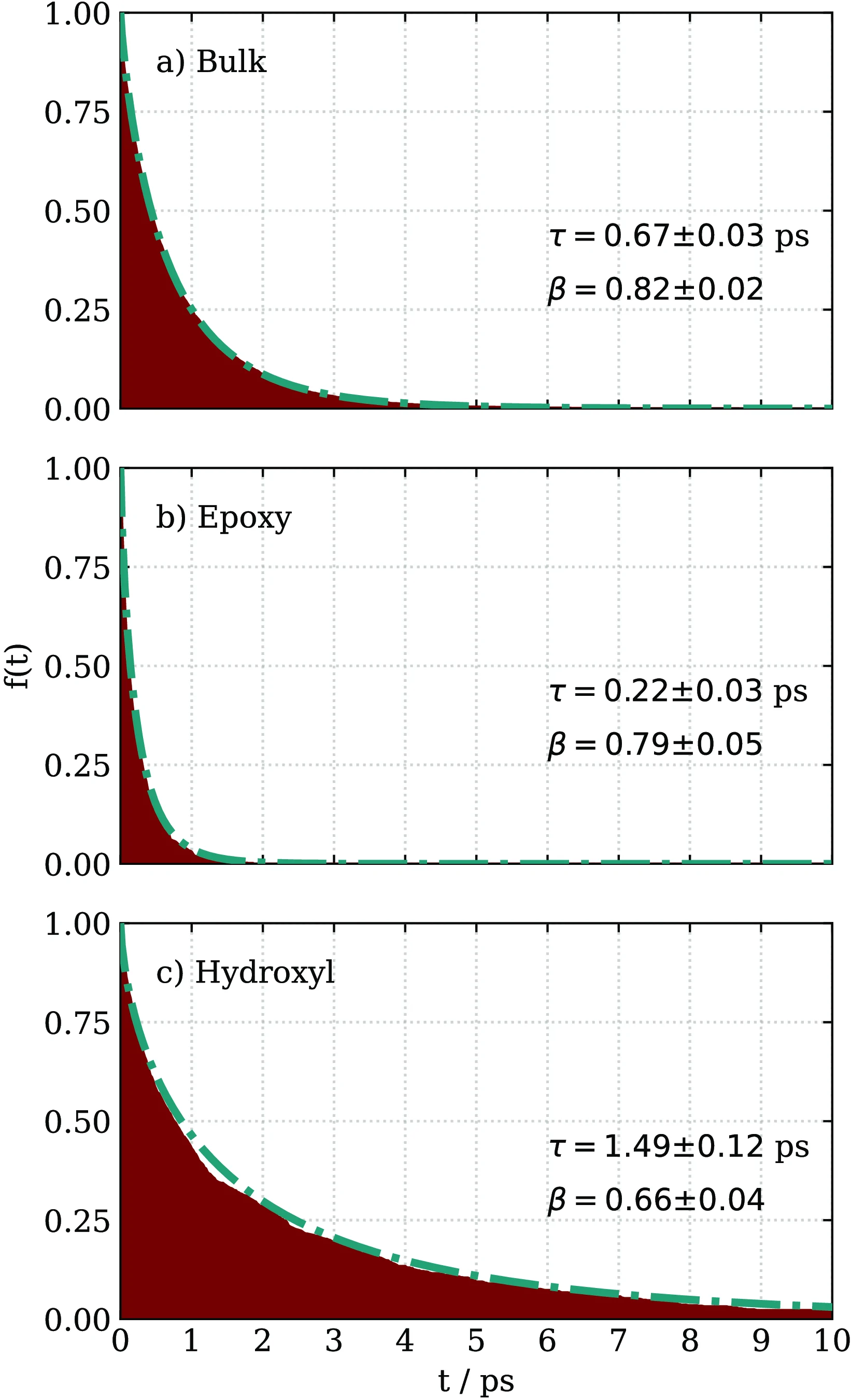
Continuous hydrogen-bond autocorrelation functions, f­(t), for (a) bulk water, (b) water molecules hydrogen-bonded to epoxy groups, and (c) water molecules hydrogen-bonded to hydroxyl groups. The shaded areas represent the autocorrelation functions obtained from the simulations, while the dashed lines correspond to stretched-exponential fits.

The characteristic relaxation times obtained for bulk water, epoxy-associated hydrogen bonds, and hydroxyl-associated hydrogen bonds are $\tau_{H_{2}O}=0.67\pm 0.03$ ps, τ_*O*_ = 0.22 ± 0.03 ps, and τ_*OH*_ = 1.49 ± 0.12 ps, respectively, while the corresponding stretching exponents are $\beta_{H_{2}O}=0.82\pm 0.02$ , β_*O*_ = 0.79 ± 0.05, and β_*OH*_ = 0.66 ± 0.04. Uncertainties represent the 95% confidence interval of the mean, calculated from 100 independent trajectories. These values indicate that hydrogen bonds involving hydroxyl groups persist significantly longer than those associated with epoxy groups, consistent with the stronger interactions revealed by the V_4S_ analysis. Conversely, hydrogen bonds formed near epoxy groups exhibit the shortest relaxation times, reflecting the comparatively weak stabilization they provide.

Values of β < 1 indicate that hydrogen-bond breaking cannot be described by a single characteristic time scale, but instead occurs through a continuous spectrum of relaxation processes. While bulk water and epoxy groups display similar stretching exponents, indicating a comparable degree of nonexponentiality, their characteristic relaxation times differ substantially. This observation implies that, despite exhibiting a similar breadth of relaxation processes, the underlying hydrogen-bond dynamics are not equivalent. Hydroxyl groups, on the other hand, simultaneously increase the characteristic relaxation time and reduce the stretching exponent. This combination indicates that hydrogen bonds formed near hydroxyl groups are not only longer-lived but also characterized by a broader distribution of relaxation times. Such behavior is consistent with the structural and dynamical picture developed throughout this work, in which hydroxyl groups generate stronger water–surface interactions, perturb the surrounding hydrogen-bond network over larger distances, and reduce the mobility of nearby water molecules. Although the microscopic origin of the broader relaxation spectrum cannot be established from the present analysis alone, the results clearly demonstrate that hydroxyl groups induce a richer and more heterogeneous dynamical environment than either bulk water or epoxy groups.

Figure 5 shows the three-dimensional MSD of all previously defined groups, with the addition of the bulk, shown in purple, for comparison. A clear difference between the epoxy and hydroxyl systems is again observed. The groups analyzed for the epoxy case (Figure 5(b)) show almost no distinction among themselves. The epoxy group does not retain nearby water molecules, on the contrary, it appears to facilitate their dynamics, allowing them to rapidly transition between groups, causing the MSD curves to become indistinguishable even at short times.

**Figure 5:**
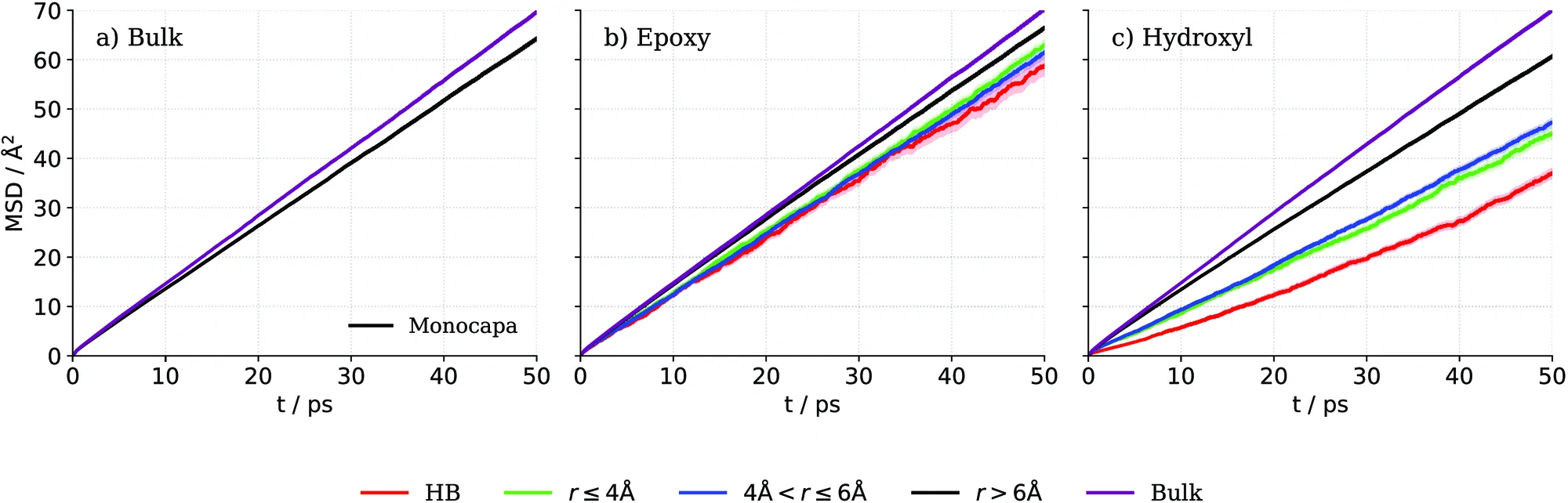
Mean square displacement of the groups defined in Figure 1, with the addition of a random selection of molecules in the bulk regime. The curves include shaded regions representing the standard error of the mean.

In contrast, the hydroxyl group (Figure 5(c)) reflects its strong interactions through long-lived hydrogen bonds, drastically reducing the mobility of the HB group compared to the others. Moreover, it also has a significant impact on the remaining groups, including molecules that do not form hydrogen bonds. A reduction in MSD is observed as the proximity to the functional group increases. This type of local effect may lead to substantial impacts on water dynamics and transport in larger systems with different degrees of oxidation and epoxy/hydroxyl ratios.^55,56^

The observed dynamics can be directly interpreted in terms of the local free-energy landscape captured by the V_4S_ index. More negative V_4S_ values correspond to energetically stable environments with stronger, longer-lived hydrogen bonds and reduced mobility, whereas higher V_4S_ values lead to weaker interactions and enhanced diffusion. This explains why epoxy-functionalized regions exhibit fast, nearly indistinguishable dynamics, while hydroxyl groups significantly slow down nearby water molecules.

To understand the origin of the differences in water interactions with the functional groups, Figure 6 presents the distributions of the Lennard-Jones and Coulomb potential contributions to the V_4S_ value separately. The red curves correspond to those shown in Figure 2 for Epoxy and Hydroxyl, included here for comparison. To compute these distributions, the interaction site corresponding to V_4S_ is first determined by calculating the total interaction energy (shown in red), and then the Lennard-Jones (shown in brown) and electrostatic (shown in yellow) contributions are computed separately.

**Figure 6:**
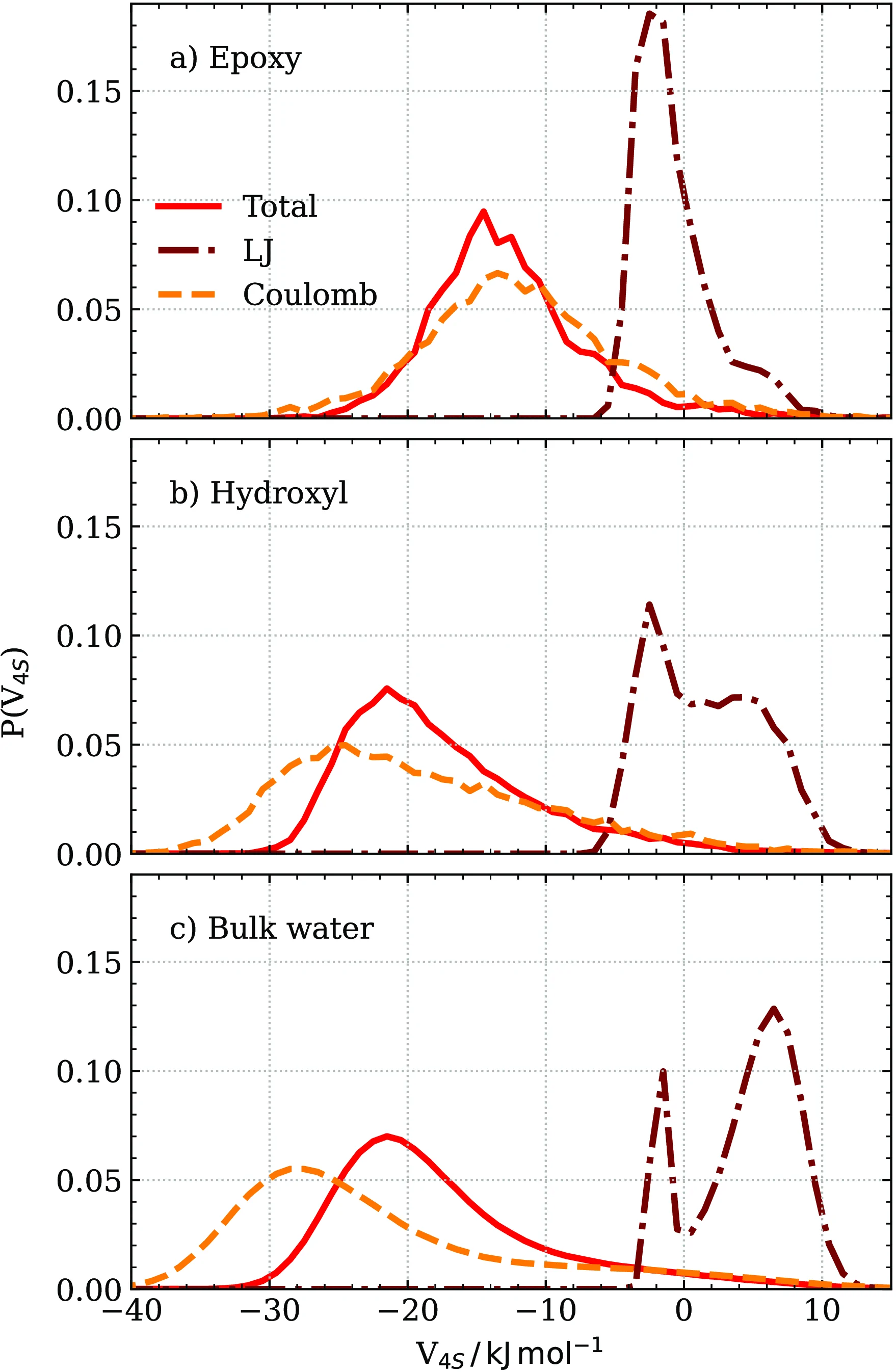
Comparison of the contributions of the Lennard–Jones (brown) and Coulomb (yellow) potentials to the distribution of the total V_4S_ (red) for different groups of water molecules. The three panels correspond to water molecules forming hydrogen bonds with (a) epoxy groups, (b) hydroxyl groups, and (c) bulk water molecules.

It is immediately evident that, in all cases, the total distribution closely resembles the Coulombic distribution. In addition, the Lennard-Jones distribution is centered around or very close to zero, indicating the dominant role of Coulomb interactions. More specifically, when comparing the distributions of water molecules forming hydrogen bonds with hydroxyl groups to those of bulk water molecules, the latter can be seen as essentially a higher-statistics version of the former. That is, the water–hydroxyl interaction is equivalent to the water–water interaction.

The bimodal Lennard-Jones (LJ) distribution observed for Hydroxyl and, more prominently, for bulk water reveals the interaction mechanism that differentiates epoxy and hydroxyl groups. The presence of a hydrogen atom in the functional group, which carries charge but has no LJ parameters, causes water molecules to experience a strong electrostatic attraction (O–H interaction). Due to the closer approach enabled by this attraction, a peak emerges between 0 and 10 kJ/mol in the hydroxyl and bulk-water cases, corresponding to the short-range repulsion of the LJ potential (∝ *r* ^–12^). However, this repulsion is still compensated by the strong electrostatic attraction generated by the charges, as reflected in the Coulombic distribution peak between −30 and −20 kJ/mol.

Finally, another aspect explored is how a water molecule approaching the graphene sheet senses the presence of a functional group. Figure 7 shows the average value of V_4S_ as a function of the distance *z* from the membranes. For this analysis, the molecules were separated into groups similar to those previously defined. However, instead of selecting only molecules in the hydration monolayer, all molecules with *z* > *z*_membrane_ were considered. The red curve represents molecules contained within a cylinder of radius 2 Å around the functional group, while the green and blue curves correspond to hollow cylindrical regions defined between 2–4 Å and 4–6 Å, respectively. The remaining molecules are represented by the black curves. The plots in the bottom row show the probability densities of finding water molecules in each of the corresponding groups. Dashed lines were added to mark local density maxima, indicating–particularly for the red group–the distance up to which the data are statistically more reliable.

**Figure 7:**
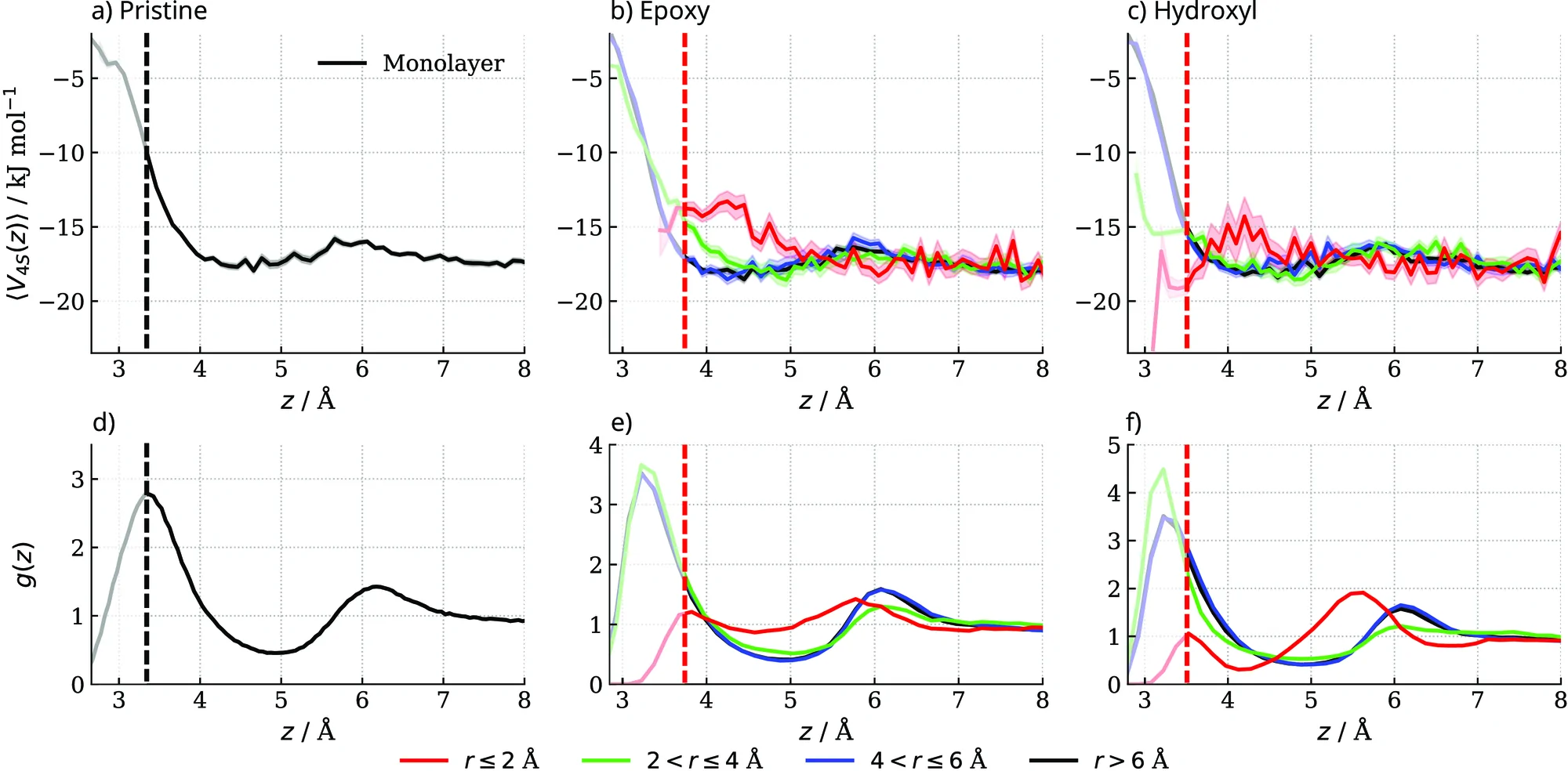
Top panels show the average value of V_4S_ as a function of *z* above the membranes, where *z* = 0 corresponds to the average position of the carbon atoms. Shaded regions represent the standard error of the mean. The bottom panels display the water density distribution along *z*. The color scheme differs slightly from previous figures: the red line corresponds to water molecules located at a radial distance $r=\sqrt{x^{2}+y^{2}}$ of up to 2 Å from the functional-group oxygen, while the green line represents molecules located between 2 and 4 Å. Red dashed lines indicate the *z* position of a *g*(*z*) maximum for the red group, while black dashed lines indicate the corresponding maximum for the black group. In the b, c, e and f panels, the black curve lies below the blue one over much of the plotted range.

The black curves in the ⟨V_4S_(*z*)⟩ plots of Figure 7 indicate the emergence of a potential barrier as a water molecule approaches the graphene surface. At the preferential interaction distance for graphene, which is most clearly observed in the pristine system, the V_4S_ value is approximately −10 kJ/mol, about 7 kJ/mol weaker than the reference value for bulk water, given by the plateau of the curve at large distances. Once again, from the blue curves in the epoxy and hydroxyl panels, which coincide almost perfectly with the black ones, we observe how local the effects produced by the functional groups are. The green curve in the Epoxy case nearly overlaps with that of the outermost molecules, however, its values begin to increase slightly earlier than the blue and black curves (with higher average values between 3.5 and 4.5 Å). For the red curve, corresponding to molecules directly above the epoxy group, a potential barrier is observed to emerge already at *z* = 5 Å. In other words, for a water molecule, being near an epoxy group is so energetically unfavorable that it would be preferable for the group not to be present at all, as the molecule would then interact more strongly with other water molecules in the hydration layer. This changes when the molecule reaches its preferential interaction site (corresponding to the peak of its *g*(*z*) in Figure 7 located close to 3.5 Å), forming a poor-quality HB of around −14 kJ/mol.

The hydroxyl group, on the other hand, exhibits a local ⟨V_4S_⟩ maximum in the region between 4.0 and 4.5 Å, followed by a pronounced minimum of ⟨V_4S_⟩ ≈ −19 kJ/mol around 3.5 Å, similar to or even lower than the ⟨V_4S_⟩ value for bulk water ( ≈−18 kJ/mol). However, when comparing this behavior with the corresponding *g*(*z*) profile, a minimum in the density is observed where a potential barrier would be expected ( ≈−19 kJ/mol). This behavior should not be interpreted strictly as a potential barrier, but rather as an indication that it is so energetically favorable for a water molecule to be located at around 3.5 Å that the region between 4 and 4.5 Å is rarely accessed. We also observe that the green curve for Hydroxyl does not follow the same trend as the others. This is attributed to the mobility of the hydrogen atom, which enables stronger interactions and the formation of hydrogen bonds with neighboring molecules, thereby extending the spatial range of the effects induced by the hydroxyl group.

In summary, the epoxy group produces interactions with water that are so weak that its incorporation into a graphene membrane disrupts the hydrogen-bond network of interfacial water molecules and destabilizes the local structure. In contrast, the hydroxyl group, due to its strong interaction with water and the presence of a hydrogen atom that enables it to act as both a donor and an acceptor of hydrogen bonds, preserves a well-structured hydrogen-bond network at the interface, effectively behaving as a water-like moiety anchored to the surface.

This scenario points to a pathway for the rational design of nanomaterials across a wide range of applications. In particular, our recent results show that there are regimes in which water structuring at interfaces can hinder the adsorption of pollutants.^15^ This highlights that, beyond the direct interaction between adsorbent and adsorbate, adsorption in aqueous environments inherently involves an additional energetic cost associated with disrupting hydration layers and creating the necessary free volume for close contact.

Within this framework, our findings suggest a route to enhance adsorption efficiency. Specifically, we show that the energetic cost of dehydration is lower for epoxy groups than for hydroxyl groups. Nevertheless, although the values differ, both groups exhibit adsorption energies for some species within the range typically associated with chemisorption (approximately – 40 kJ/mol).^57^ Pristine graphene, in contrast, is characterized by adsorption energies in the physisorption regime and by a highly structured hydration layer, both of which disfavor adsorption. Altogether, these results emphasize that optimal adsorbent design must balance not only adsorbate–surface affinity but also the ability to locally disrupt the hydrogen-bond network of water, thereby minimizing the free-energy penalty associated with dehydration.

More broadly, these results indicate that interfacial hydration in graphene oxide is intrinsically heterogeneous at the nanoscale, with distinct functional groups creating regions of markedly different energetic and dynamical character. The overall behavior of the system should therefore be understood as the result of a spatially heterogeneous landscape rather than a uniform interfacial environment.

## Conclusions

In this work, we have presented a microscopic, energetically grounded characterization of water–surface interactions in graphene-based systems functionalized with epoxy and hydroxyl groups. Employing the V_4S_ structural index, we were able to resolve, with spatial and energetic specificity, how distinct chemical functionalities modulate the hydrogen-bond (HB) network of interfacial water. This approach moves beyond traditional density-based or geometric descriptors, providing instead a direct link between local structure and interaction energetics.

Our results reveal a clear and physically consistent dichotomy between epoxy and hydroxyl groups. Epoxy functionalities, despite satisfying standard geometric HB criteria, give rise to energetically weak interactions that fail to compensate for the disruption of the bulk water network. As a consequence, they generate only weakly hydrophilic local environments, characterized by defective hydration structures, reduced HB lifetimes, and enhanced molecular mobility. In contrast, hydroxyl groups behave effectively as water-like moieties anchored to the surface: they sustain strong, long-lived hydrogen bonds with surrounding molecules, preserve the integrity of the tetrahedral network, and significantly slow down local dynamics. In particular, the behavior of epoxy groups reveals that chemical polarity alone is not sufficient to ensure strongly hydrophilic behavior, as energetically weak interactions may still result in only limited local compensation of hydrogen-bond defects.

A key outcome of this study is the demonstration that these effects are highly localized, decaying within a few angstroms from the functional site. However, despite their short spatial range, their impact on both structure and dynamics is profound. In particular, we find that local chemical functionalization can induce nonlocal perturbations in the hydration structure, but only when the surface interaction is sufficiently strong to compensate for hydrogen-bond defects: this behavior is clearly observed for hydroxyl groups, whereas epoxy functionalities remain essentially local. This highlights the importance of nanoscale chemical patterning in determining macroscopic properties such as wettability, permeability, and adsorption.

From a broader perspective, the present results support a unifying interpretation of hydrophilicity and hydrophobicity in terms of the ability of a surface to energetically compensate for HB defects induced in water. Within this framework, the V_4S_ index emerges as a powerful, parameter-free descriptor capable of quantitatively capturing this balance at the molecular level. While this definition is grounded in the fundamental thermodynamics of hydrogen-bond network perturbations, its universality is currently validated for a variety of extended aqueous interfaces, charged systems, proteins and biological membranes, which test the robustness of the defect-compensation paradigm in increasingly complex physical environments. Its applicability across bulk, interfacial, and nanoconfined environments positions it as a valuable tool for the rational design of materials where water plays a central role. Even more broadly, these results indicate that interfacial hydration in graphene oxide is intrinsically heterogeneous at the nanoscale, with different functional groups generating regions of distinct energetic and dynamical character.

The implications of these findings extend to a wide range of applications, including membrane-based separations, desalination, and adsorption processes. In particular, the contrasting behavior of epoxy and hydroxyl groups suggests distinct functional roles: while hydroxyl groups contribute to stabilizing structured hydration layers, epoxy groups may promote dehydration and facilitate access to reactive sites, potentially enhancing adsorption efficiency. The ability to tune the relative abundance and spatial distribution of these groups, therefore, offers a concrete pathway to engineer graphene-based materials with tailored interfacial properties.

Overall, this work provides both conceptual and methodological advances in understanding water at complex interfaces. By establishing a direct connection between local energetics, structure, and dynamics, it lays the groundwork for a predictive framework of interfacial hydration, with potential impact across soft matter, biophysics, and nanotechnology. Ultimately, our results show that hydrophilicity is an emergent property governed by local energetic balance, rather than a direct consequence of surface chemistry alone.

## Supplementary Material



## Notes

Additional V_4S_ probability distributions obtained using the TIP4*P*/2005 water model, comparisons between real-dynamics and inherent-structure configurations (PDF)
