# Supplementary material for: Hydrophilicity as Compensation of Hydrogen-Bond Defects at Graphene Oxide Interfaces
Source: ACS Nanosci Au. 2026 Jul 20;6(5):885–94. doi: 10.1021/acsnanoscienceau.6c00063 (PMC13693188; doi:10.1021/acsnanoscienceau.6c00063)
Supplement: Supplementary file 1 [file ng6c00063_si_001.pdf]

Hydrophilicity as Compensation of Hydrogen-Bond Defects at Graphene Oxide Interfaces

Patrick R. Bredow Côrtes[1,2], Nicolás A. Loubet[2], Mateus H. Köhler[3], José Rafael Bordin[1,4], and Gustavo A. Appignanesi[2]

[1]Departamento de Física, Instituto de Física e Matemática, Universidade Federal de Pelotas, Pelotas, Brazil
[2]INQUISUR, Departamento de Química, Universidad Nacional del Sur (UNS)-CONICET, Argentina
[3]Departamento de Física, Universidade Federal de Santa Maria, 97105-900 Santa Maria, Brazil
[4]Fachbereich Physik, Universität Konstanz, Konstanz, Germany

Supporting Information

Corresponding authors:
Mateus H. Köhler: mateus.kohler@ufsm.br
José Rafael Bordin: jrbordin@ufpel.edu.br
Gustavo A. Appignanesi: appignan@criba.edu.ar

Robustness with Respect to the Water Model

Previous studies introducing and validating the V_{4S} framework have shown that the structural classification provided by this descriptor is largely independent of the particular water model employed [1, 2]. Here, we implement the TIP4P/2005 model [3] and compare the resulting V_{4S} distributions with those obtained for the SPC/E model to further explore the transferability of the physical interpretation associated with V_{4S} across different water descriptors.

Figure S1 presents the normalized probability distributions of V_{4S} obtained from inherent structure (IS) configurations for water molecules classified according to their interaction with the graphene oxide functional groups. The overall behavior closely resembles that reported in the main text for SPC/E water. In particular, water molecules forming HBs with hydroxyl groups display distributions shifted toward lower V_{4S} values, whereas molecules hydrogen-bonded to epoxy groups populate significantly less favorable regions of the distribution.

Importantly, the characteristic positions of the distribution maxima remain essentially unchanged with respect to the SPC/E results. The clear separation between hydroxyl- and epoxy-associated hydrogen-bonded molecules is therefore preserved across water models, indicating that the observed effect is not a model-dependent artifact but rather a robust feature of the interfacial hydrogen-bond network.

A more quantitative comparison can be obtained by analyzing the fraction of hydrogen-bonded molecules exhibiting highly favorable local environments, defined here by $V_{4S} < -20$ kJ mol^{-1}. For molecules forming HBs with hydroxyl groups, 50.5% of TIP4P/2005 molecules satisfy this condition, compared with 44.5% for SPC/E. In contrast, only 9.9% and 5.5% of molecules hydrogen-bonded to epoxy groups reach such low V_{4S} values for TIP4P/2005 and SPC/E, respectively. These results confirm that hydroxyl groups preferentially stabilize local water environments associated with a lower concentration of hydrogen-bond defects, whereas

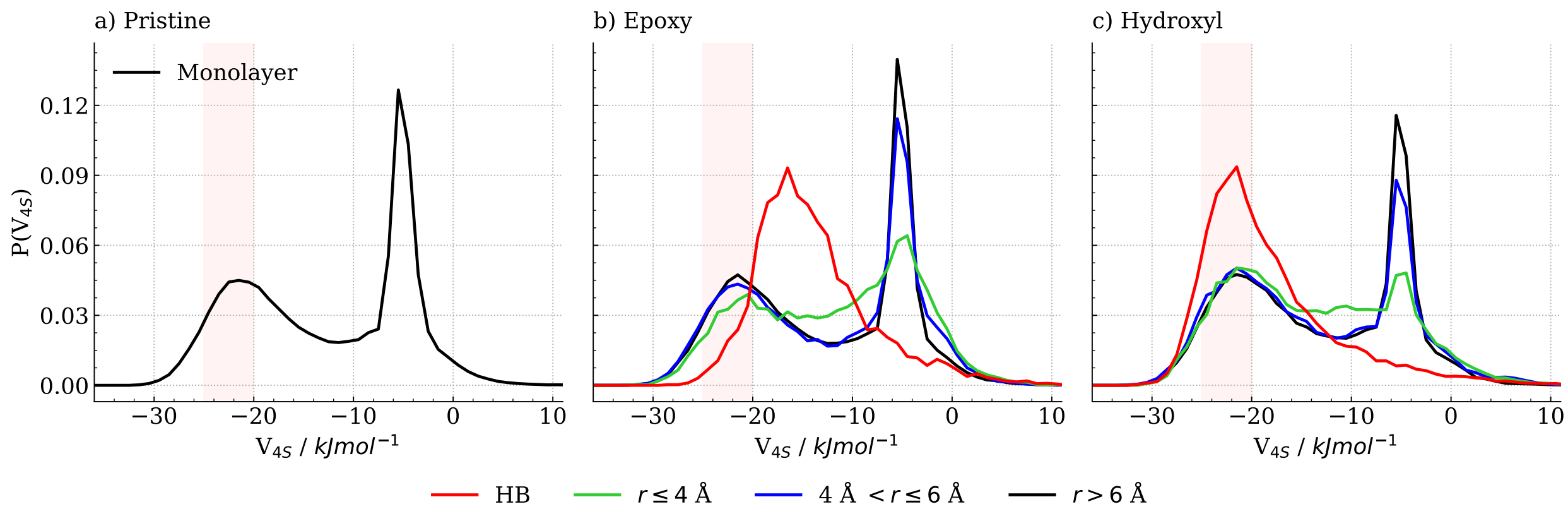

Figure S1: Normalized probability distribution of V_{4S} values calculated from inherent structure (IS) configurations of the TIP4P/2005 water model. Water molecules are classified according to the same criteria employed in the main text. The separation between hydroxyl- and epoxy-associated hydrogen-bonded molecules closely resembles that obtained for SPC/E water.

epoxy groups are considerably less effective in promoting such favorable local structures. These results do not depend on the water model.

Taken together, these findings demonstrate that the central conclusions of the present work are insensitive to the particular molecular description of water employed. The compensation mechanism discussed in the main text, whereby hydrophilic functional groups locally recover hydrogen-bond connectivity disrupted by the graphene substrate, emerges consistently in both water models.

Analysis of Real Dynamics Configurations

Throughout the main text, the V_{4S} analysis was performed on inherent structure configurations obtained by energy minimization of instantaneous molecular dynamics snapshots. Previous studies have shown that this procedure significantly enhances the ability of V_{4S} to identify local structural environments by removing thermal fluctuations that obscure the underlying organization of the hydrogen-bond network [1, 2].

To illustrate this point in the present system, Figures S2 and S3 show the corresponding V_{4S} distributions obtained directly from real dynamics (RD) configurations for SPC/E and TIP4P/2005 water, respectively.

Compared with the inherent-structure distributions discussed in the main text, the distinctions between the different molecular populations become substantially less pronounced. Thermal fluctuations broaden all distributions and strongly reduce the separation between molecules interacting with hydroxyl and epoxy groups. As a consequence, assigning molecules to distinct local environments based solely on instantaneous configurations becomes considerably more difficult.

Nevertheless, an important signature of the effect survives even in the presence of thermal noise. In both water models, the distributions associated with hydrogen-bonded molecules remain slightly shifted relative to each other, with the hydroxyl-related peak appearing at approximately 4 kJ mol^{-1}) lower V_{4S} values than the corresponding epoxy peak. Although modest, this residual displacement indicates that the structural differences identified in the inherent structures are already encoded in the instantaneous liquid configurations.

Therefore, the minimization procedure does not generate the observed distinction between hydroxyl and epoxy environments. Instead, it acts as a filter that removes vibrational and thermal contributions, revealing the underlying energetic landscape with much greater clarity. The persistence of the same qualitative trend in both RD and IS analyses reinforces the interpretation that the different functional groups induce genuinely distinct local hydrogen-bond organizations in the surrounding water.

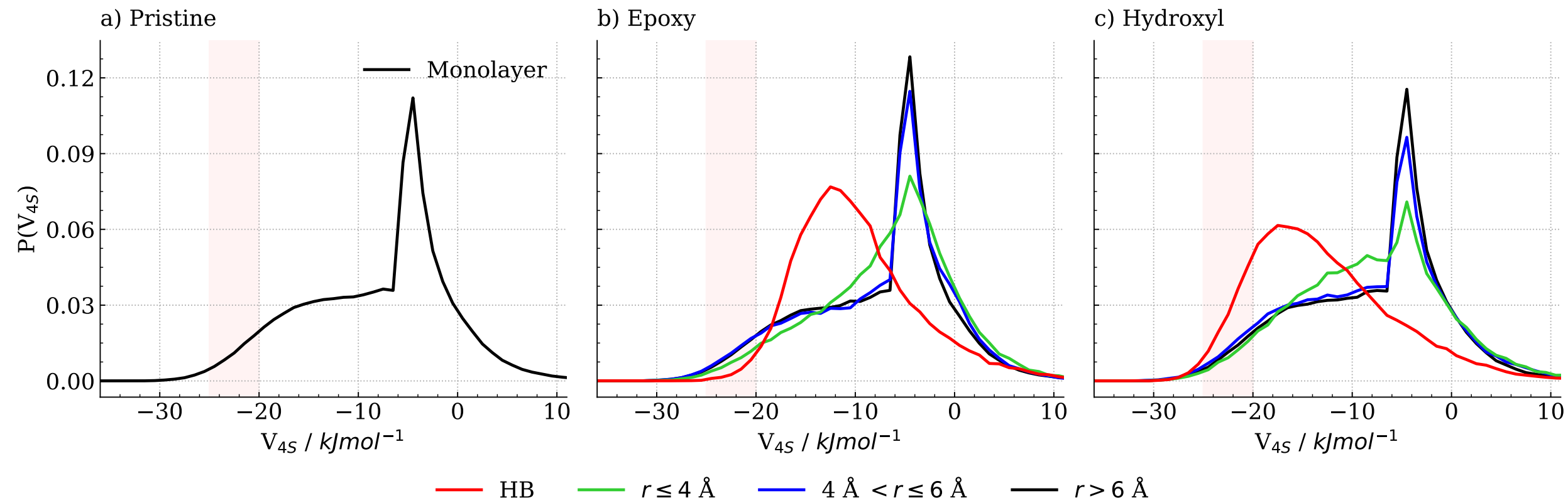

Figure S2: Normalized probability distribution of $V_{4\mathrm{S}}$ values calculated from real dynamics (RD) configurations of the SPC/E water model. Thermal fluctuations broaden the distributions and partially mask the separation observed in inherent structures, although a small shift between hydroxyl- and epoxy-associated hydrogen-bonded molecules remains visible.

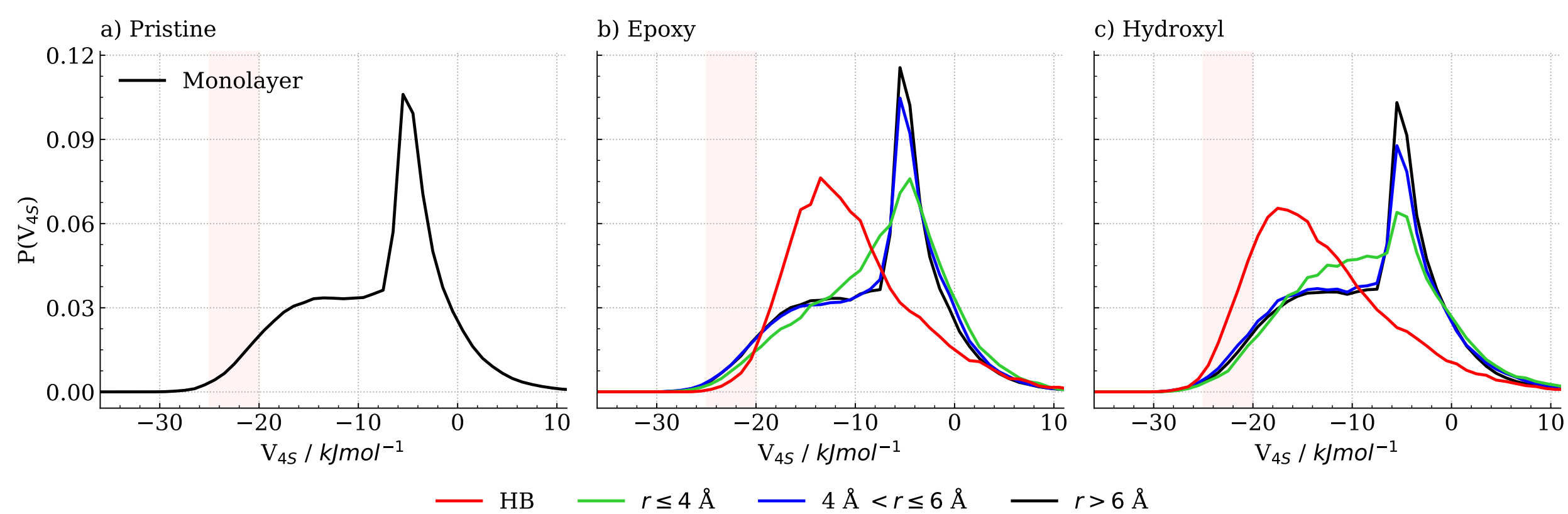

Figure S3: Normalized probability distribution of $V_{4\mathrm{S}}$ values calculated from real dynamics (RD) configurations of the TIP4P/2005 water model. As in SPC/E water, the differences between molecular populations are substantially reduced relative to the inherent-structure analysis but remain qualitatively detectable.

References

(1) Loubet, N. A.; Verde, A. R.; Appignanesi, G. A. *The European Physical Journal E* **2024**, *47*, 61.

(2) Loubet, N. A.; Verde, A. R.; Appignanesi, G. A. *The Journal of Chemical Physics* **2024**, *160*.

(3) Abascal, J. L. F.; Vega, C. *The Journal of Chemical Physics* **2005**, *123*, 234505.
